# Exploring Sentence Diversity at the Boundary of Typical and Impaired Language Abilities

**DOI:** 10.1044/2020_JSLHR-20-00031

**Published:** 2020-10-15

**Authors:** Pamela A. Hadley

**Affiliations:** a Department of Speech and Hearing Science, University of Illinois at Urbana–Champaign

## Abstract

**Purpose:**

This review article summarizes programmatic research on sentence diversity in toddlers developing language typically and explores developmental patterns of sentence diversity in toddlers at risk for specific language impairment.

**Method:**

The first half of this review article presents a sentence-focused approach to language assessment and intervention and reviews findings from empirical studies of sentence diversity. In the second half, subject and verb diversity in three simple sentence types are explored in an archival database of toddlers with varying levels of grammatical outcomes at 36 months of age: low average, mild/moderate delay, and severe delay.

**Results:**

Descriptive findings from the archival database replicated previous developmental patterns. All toddlers with low-average language abilities produced diverse simple sentences by 30 months of age and exhibited greater sentence diversity with first-person *I*-subjects before third-person subjects. Third-person subject diversity emerged in a developmental sequence, increasing in one-argument copula contexts and one-argument subject–verb sentences before two-argument subject–verb–object sentences. This developmental pattern held across all three outcome groups. Third-person subjects were least diverse for children with severe grammatical delays and were absent in all sentence contexts for two children with severe delays at 36 months.

**Conclusions:**

Sentence diversity increases gradually and expands in predictable patterns. Understanding these developmental patterns may help identify and treat children who display unexpected difficulty combining different subjects and verbs in flexible ways.

**Supplemental Material and Presentation Video:**

https://doi.org/10.23641/asha.12915320

A hallmark characteristic of preschool children with specific language impairment (SLI) is difficulty with the acquisition of grammar, even when compared to younger typically developing children matched for mean length of utterance (MLU; cf. Leonard, 2014; Schwartz, 2017). Although a considerable body of research has focused on the protracted mastery of obligatory tense and agreement morphemes (cf. Oetting & Hadley, 2017, for a review), English-speaking preschool children with SLI show other indicators of difficulty with sentence structure. For example, toddlers at risk for SLI have late onset and slower growth of the verb lexicon (Ellis Weismer et al., 2001; Hadley et al., 2016; Olswang et al., 1997) and difficulty learning and producing verbs compared to typically developing peers in the preschool years (Conti-Ramsden & Jones, 1997; Fletcher & Peters, 1984; Rice et al., 1994; Watkins et al., 1993). English-speaking preschoolers with SLI are also more likely to omit sentence subjects (Fletcher et al., 2012; Grela & Leonard, 1997) and to make more pronoun case errors in subject position relative to language-matched peers (Loeb & Leonard, 1991; Moore, 1995; Wexler et al., 1998). In short, young children with SLI have difficulty with the essential building blocks of grammar*—*specifically, the subjects and verbs that make up simple sentences.

The purpose of this review article is to summarize programmatic research on sentence diversity and to describe how basic sentence structure emerges in the language of toddlers at risk for SLI. The review article is organized in two parts. The first half provides an overview of the sentence-focused framework, measures of sentence diversity, and empirical findings from studies of sentence diversity with toddlers developing typically and those at risk for SLI. The second half presents descriptive results from an exploratory study of subject diversity, revealing predictable developmental patterns and characteristics associated with the children at greatest risk for persistent SLI. The review concludes with a discussion of the clinical implications, limitations of the research program, and directions for future research.

## An Overview of the Sentence-Focused Framework

The sentence-focused framework was developed to highlight theoretical and empirical links between early- and later-developing elements of English sentence structure (cf. Hadley, 2006, 2014). The framework is intended to bridge early vocabulary and grammar interventions for toddlers and preschoolers. By understanding how grammatical structures are related to one another and prioritizing treatment targets accordingly, the efficiency of grammar interventions may be improved. The framework is conceptualized as a series of four developmental steps: words, verbs, childlike sentences, and adult sentences. The first two steps, words and verbs, focus on building a core vocabulary and a diverse verb lexicon. Childlike sentences are placed on the third step. Childlike sentences have a subject and a lexical verb. These sentences are characterized by omission of grammatical morphemes and developmental errors. Adult sentences are placed on the final step. Adult sentences are augmented by language-specific grammatical structures such as tense and agreement marking in English, which operate with scope over basic sentence structure.

The second step of the framework recognizes the unique role of verbs in building sentence structure. Verbs carry information about the number of participants in an event and the semantic roles of those participants (Fletcher & Frizelle, 2017; Haegeman, 1994; Pinker, 1989; Reinhart, 2003; Schwartz et al., 2017). This is known as a verb's argument structure. Argument structure dictates how a sentence is built. The sentences in (1)–(4) illustrate this. The verb *play* in (1a) requires only one argument, an animate agent, whereas the verb *build* in (1b) requires two participants, one to cause the action and an affected object to receive that action. Verbs that require only one argument are called intransitive verbs. Intransitive verbs are the basis of subject–verb (SV) sentences. Verbs that require two arguments are called transitive verbs. Transitive verbs are the basis of subject–verb–object (SVO) sentences. A fair number of verbs can be both intransitive and transitive. Examples of two common alternation types are shown in (2) and (3). The sentence pairs for *drink* in (2a) and (2b) have the same subject *baby*. In contrast, *cake* is the subject of intransitive *bake* in (3a), whereas *Mom* is the subject of transitive *bake* in (3b). The same “verb” form can also have different meanings associated with different argument structures as in (4). Intransitive *get* in (4a) refers to a change of state; the pizza *became* hot. In contrast, transitive *get* in (4b) has a causal meaning; the girl caused the *possession* of the snack. These examples illustrate how complicated verb learning can be. Verbs cannot be learned without their argument structure.

(1a) The boy is *playing*.  = 1 argument, intransitive, SV

(1b) The boy is *building* a tower. = 2 arguments, transitive, SVO

(2a) The baby is *drinking*.  = 1 argument, intransitive, SV

(2b) The baby is *drinking* juice. = 2 arguments, transitive, SVO

(3a) The cake is *baking*.  = 1 argument, intransitive, SV

(3b) Mom is *baking* a cake.  = 2 arguments, transitive, SVO

(4a) The pizza got hot.  = 1 argument, intransitive, SV

(4b) The girl *got* a snack.  = 2 arguments, transitive, SVO

Verbs also express how events unfold in time and how entities relate to one another within events (Ramchand, 2008). The sentences in (5) show how different semantic properties of verbs influence the encoding of tense and aspect in adult sentences (cf. Rispoli et al., 2012). Notice that the action verb *sleep* in (5a) is associated with auxiliary *is* and –*ing* to mark present tense and progressive aspect, respectively, whereas the state verb *need* in (5b) is associated with the verb inflection –*s* to encode simple present tense.

(5a) The baby is *sleeping*.   = action verb, auxiliary *is* + –*ing*

(5b) The bab*y needs* a blanket.  = state verb, –*s*

The sentence-focused framework predicts that children's early verb vocabulary will be more strongly associated with later grammatical outcomes than early noun vocabulary. Hadley et al. (2016) tested this hypothesis in a study with 45 typically developing English-speaking toddlers. Noun and verb diversity were assessed by parent report on the MacArthur–Bates Communicative Development Inventories (CDI; Fenson et al., 2007) and from the number of different nouns and verbs used spontaneously in parent–child language samples. They found that 24-month measures of verb diversity in comparison to measures of noun diversity were better predictors of 30-month grammar outcomes as measured by the Index of Productive Syntax (IPSyn; Scarborough, 1990). This study explored only the contribution of early vocabulary to later grammar and not the reverse, given that single-word utterances appear before sentence structure begins to develop. However, it should be noted that children are learning words and grammar simultaneously and in interdependent ways.

Sentence diversity is emphasized on the third step of the framework, childlike sentences. Sentence diversity lays the foundation for the emergence and growth of tense and agreement morphemes in English (cf. Hadley, Rispoli, & Holt, 2017; Rispoli & Hadley, 2011). Diverse subjects, distributed across the grammatical features of person and number, provide children with opportunities to encode SV agreement with different surface forms (e.g., *I am* vs. *the penguin is; the blocks are*). Diverse verbs provide them with opportunities to encode the interaction between aspect and tense (e.g., *is eating* vs. *tastes good*; Rispoli et al., 2012). Sentence diversity is operationalized as the number of unique SV combinations (see Hadley et al., 2018, for a clinically oriented tutorial). Developmentally, sentences with first-person subjects precede sentences with third-person subjects, and singular subjects precede plural subjects (Lee, 1974). Children's ability to produce diverse sentences reflects the increasing strength of their grammatical knowledge. As sentences become more diverse, it is more likely that children are using grammatical knowledge to produce sentences rather than relying on rote, memorized chunks. The sentence-focused framework predicts that exposure to diverse noun subjects in the adult input will strengthen children's underlying representation of sentence structure later in development (Hadley, Rispoli, & Holt, Papastratakos, et al., 2017). Studies documenting increases in sentence diversity with age and grammatical development and empirical links between growth in sentence diversity and tense and agreement are reviewed below.

## Sentence Diversity as a Complementary, Developmental Measure

Although difficulty with the acquisition of sentence structure is a core diagnostic feature of language disorders (American Psychiatric Association, 2013), existing clinical analysis methods do not adequately characterize children's ability to combine different subjects and verbs into sentences in flexible ways. This is especially important because sentence diversity is a precursor to the later diagnostic marker of tense and agreement in English. MLU (Brown, 1973) remains the primary measure of grammatical development for children under 3 years of age (cf. Tager-Flusberg et al., 2009). Although MLU increases as new morphological and syntactic structures appear in children's spontaneous speech, MLU is a global measure of utterance length. It does not inventory the presence of specific syntactic structures nor the lexical diversity within those structures. As such, MLU cannot reveal limited flexibility with SV combinations or an overreliance on formulaic sentence frames (e.g., *I want X; that*'*s a X*) in toddlers at risk for SLI (e.g., Hadley, 2014; McKenna & Hadley, 2014). In contrast, comprehensive grammatical analysis approaches such as the Language Assessment, Remediation and Screening Procedure (Crystal et al., 1989) and the IPSyn quantify the presence or absence of specific morphological and syntactic structures that emerge between 1.5 and 5 years of age from language samples. These measures cover broad developmental periods and are appropriate for characterizing the severity of a language disorder. Although the IPSyn is used widely in research with toddlers, this comprehensive measure does not adequately reflect diversity in early sentences either. To illustrate, only two of 56 items on the IPSyn examine the simple sentences SV and SVO. Items are scored as 0 if a structure is absent, 1 for one example, or 2 for two sufficiently different examples. The IPSyn's scoring rules “also credit” children for simpler structures if more advanced ones are apparent. Therefore, two sufficiently different instances of SVO sentences will “also credit” SV sentences. This means a child who produces potentially formulaic utterances such as *I did it* and *I want that* would receive all four points for SV and SVO sentences, even though there may be no evidence of SV sentences. Thus, the IPSyn is not designed to reveal differences between children in the diversity of SV and SVO sentences.

**Figure 1.**
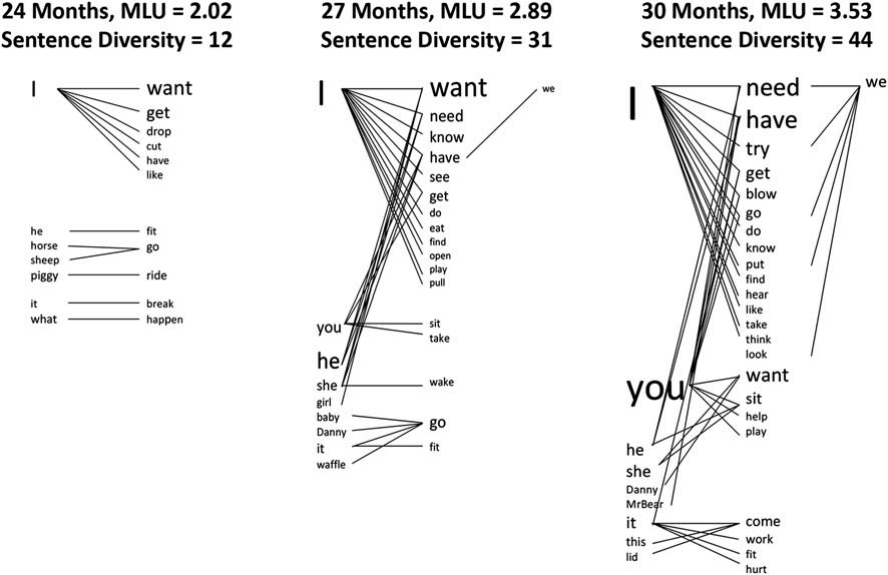
Developmental change in sentence diversity. Each line represents a unique subject–verb combination. Font sizes for each subject and verb reflect the frequency of use for each word (larger font indicates more frequent use). MLU = mean length of utterance. Adapted from Hadley et al., 2018.

To characterize developmental differences in early sentence development and children's ability to use basic sentence structure in lexically flexible ways, we developed a set of sentence diversity measures. The measures reflect the number of different words produced in structurally defined positions of early sentences. Subject diversity is operationalized as the number of different pronouns, proper nouns, and head nouns in the subject noun phrase (NP) position. Verb diversity is operationalized as the number of different verbs in the head verb position of a main clause. All inflectional forms of a verb (regular and irregular) are counted as a single type. Sentence diversity is operationalized as the number of unique combinations of a subject and a lexical verb (Hadley, Rispoli, & Holt, Papastratakos, et al., 2017; see Hadley et al., 2018). Subject diversity, verb diversity, and SV diversity are simple counts of each different word and each unique combination. Figure 1 illustrates developmental change in sentence diversity for a child with an average MLU of 2.02 at 24 months of age. The font size increases with the number of times the child used a given subject or verb. The lines indicate the number of unique SV combinations produced by the child. As can be seen, sentence diversity increases as the number of different subjects and verbs increases. Between 24 and 30 months, subject diversity increased from seven to 10 different subjects, verb diversity increased from 11 to 23 different verbs, and sentence diversity increased from 12 to 44 as the child began to combine verbs with more different subjects. At 30 months, this child's MLU and sentence diversity score are both in the above-average range.

Measures of sentence diversity are intended to complement measures of MLU and grammatical complexity, not to replace them. They are also most appropriate for the developmental period in which children transition from word combinations to sentences, corresponding to MLUs from 1.50 to 2.50. In the studies reviewed below, sentence diversity has been computed from language samples during free-play using a standard set of toys in a lab-based playroom. The measures are rate based, computed from utterances produced in a fixed length of time (e.g., 30 min) rather than from a specific number of utterances, to capture differences in children's ability to translate diverse messages into sentences in real time.

To establish developmental expectations for sentence diversity, we assessed sentence diversity for 40 typically developing monolingual English-speaking children at 30 months of age from 30-min parent–child language samples (Hadley et al., 2018). Children were drawn primarily from White, non-Hispanic college-educated homes. On average, children produced more than 200 complete and intelligible utterances in 30 min (*M* = 215.48, *SD* = 70.74), but only 27% of these utterances were sentences (*M* = 58.83, *SD* = 36.10). Average sentence diversity was 28.23 (*SD* = 13.83), approximately one unique SV combination per minute. All 40 toddlers also produced 10 or more simple sentences and had at least two SV combinations of both first- and third-person singular subjects. Sentence diversity at 30 months was also positively associated with MLU and grammatical complexity as measured by the IPSyn, providing additional support for sentence diversity as a valid and complementary developmental measure. In short, sentences increased in diversity as utterances became longer and more complex.

## Empirical Studies of Sentence Diversity

Initially, we conceptualized sentence diversity broadly, recognizing how different verbs and different subjects create diverse sentences. These studies revealed a developmental sequence in the expansion of subject types. They also provided evidence of the role third-person sentences play in grammatical development for English-speaking preschoolers. More recently, we have focused on sentences with third-person subjects, given the hypothesized link between this subject type and the emergence of tense and agreement in English (Rispoli et al., 2012).

To begin, Hadley et al. (2018) related patterns of subject type expansion to children's levels of grammatical development by grouping the 40 children into quartiles by MLU. The average MLUs for the quartiles were 2.13, 2.66, 3.04, and 3.76, corresponding to Brown's (1973) Stages II, III, IV, and V, respectively. Diverse sentences were observed primarily with first-person singular *I*-subjects^1^ and third-person singular subjects, with fewer instances of second-person and plural subjects. Moreover, the number of different sentences with *I*-subjects was relatively similar across the four MLU groups. Thus, differences in sentence diversity at 30 months were largely attributable to SV combinations with third-person subjects. Further inspection of the subject types revealed that pronoun subjects were more common than noun subjects. All 40 children produced *I*-subjects; 35 children produced *you-*subjects; and approximately two thirds of the children produced *we*-*, he*-*,* and *it*-subjects at 30 months. In contrast, noun subjects were limited. Additionally, animate noun subjects (e.g., *baby, cow, farmer*) were more common than inanimate noun subjects (e.g., *bottle, food, tractor*). Because the patterns of subject type expansion were not constrained by children's ability to combine words or the availability of words in their expressive vocabularies, we interpreted the qualitative patterns as developmental differences in syntactic ability. We concluded that toddlers who produced *I-*sentences exclusively had less advanced syntactic development than toddlers who produced diverse third-person sentence subjects.

Second, we have developed input modification strategies to promote children's sentence diversity. In a quasi-experimental study, Hadley, Rispoli, Holt Papastratakos, et al., (2017) examined changes in noun subject diversity in parent input following instruction in toy talk and then determined if noun subject diversity in parent input facilitated toddlers' use of diverse third-person sentences. Toy talk was taught to parents in the context of general responsive interaction and descriptive language modeling strategies. The first strategy—*talk about the toys—*was intended to increase third-person sentences about concrete objects in the play environment. The second strategy—*give the object its name—*was designed to increase parents' noun subjects. Based on the findings of Thompson and Newport (2007), we hypothesized that noun subject diversity in parent input would alter the statistical distribution of cues that signal the boundary between subject and predicate constituents. This would, in turn, strengthen the representation of clause structure in children's underlying grammar. All dyads (*n* = 38) were monolingual speakers of English. Parents were primarily White, non-Hispanic, and college educated. All toddlers were typically developing with language abilities in the low-average range. Toy talk instruction occurred when toddlers were between 21 and 24 months of age. Toddlers' sentence diversity with third-person subjects was assessed every 3 months from 21 to 30 months from 1 hr of conversational language samples. Following toy talk instruction, parents in the treatment group increased the diversity of noun subjects in declarative sentences (e.g., *Your castle is wobbling.*) compared to parents in a quasi-control group. Across both groups, parents' number of different noun subjects ranged from one to 40, and this variable was a significant predictor of toddlers' growth in sentence diversity with third-person subjects. Although the quasi-experimental nature of the design requires cautious interpretation of the findings, strategies that shift conversations toward the third-person discourse space and increase the diversity of noun subjects in input hold promise for promoting children's flexible use of third-person sentences.

Third, Hadley, Rispoli, and Holt, et al. (2017) conducted a follow-up study to determine whether children's growth in sentence diversity with third-person subjects was a significant predictor of growth in the productivity of tense and agreement morphemes. A morpheme's productivity is characterized by the number of different contexts it appears in, such as the number of different subjects appearing with copula *is* or the number of different verbs inflected with past tense –*ed*. The more productive a morpheme is, the more likely it exists as a separate part of grammatical knowledge and not simply as part of an unanalyzed word or phrase. Thus, diverse sentence contexts are necessary for demonstrating productive use of tense and agreement morphemes.

Tense/agreement productivity (TAP) scores (Hadley & Short, 2005) were computed and modeled for the same 38 children using the 1-hr language samples obtained at 21, 24, 27, and 30 months. TAP growth was centered at 30 months. For typically developing toddlers, an average 30-month TAP score is approximately 10 (Hadley et al., 2014), or 10 uses of tense/agreement morphemes across diverse sentence contexts. Significant TAP growth remained evident even after controlling for children's growth in sentence diversity, and growth in sentence diversity was a significant predictor of TAP growth over time. With every five unique third-person SV combinations, TAP scores increased by 1 point. This finding indicates that children's knowledge of clause structure, as indexed by a measure of sentence diversity with third-person subjects, contributes to developmental changes in the marking of tense and agreement.

Finally, McKenna and Hadley (2014) conducted a retrospective analysis of sentence diversity at 30 months for two English-speaking children identified as at risk for SLI based on below-average criterion scores on the Test of Early Grammatical Impairment at 36 months (Rice & Wexler, 2001). At 30 months, the children had low expressive vocabularies at the 10th and 20th percentiles, but they were combining words on a regular basis with MLUs of 2.62 and 1.78. The children's sentence diversity was limited in different ways. One child produced *I*-sentences with 15 different verbs. However, there was a stark imbalance in the total number of sentences with first-person singular subjects (62) to other subject types (4). The majority of *I*-sentences were combined with the verb *want* in both simple (e.g., *I want milk*) and complex sentences with two or three verbs (e.g., *I wanna do that; I wanna try chicken going in*). This child produced only two simple sentences with third-person subjects (e.g., *and that*'*s go top; they eat some more pizza*). The second child produced only seven different SV combinations in total and made argument structure errors when producing third-person subjects (e.g., *and chicken make; a knife stir*). We proposed that limited sentence diversity at 30 months, with third-person subjects in particular, may foreshadow later problems with tense/agreement marking. Clearly, further documentation of the emergence of sentence diversity in toddlers at risk for SLI is warranted to determine if limited sentence diversity elevates children's risk for persistent SLI.

This program of research has established links between sentences with third-person subjects and tense/agreement marking. Collectively, our studies with typically developing, English-speaking toddlers have revealed that (a) sentence subjects expand in a developmental sequence with sentences with first-person subjects increasing in diversity before sentences with third-person subjects, (b) subject diversity in parent input promotes children's growth of sentence diversity with third-person subjects, and (c) children's growth in sentence diversity with third-person subjects is a significant predictor of growth in tense/agreement morphemes. From a clinical perspective, these findings provide a developmental rationale for targeting sentences with third-person subjects as part of early grammatical intervention (cf. Hadley et al., 2018). However, little is known about the emergence of third-person subjects to guide intervention efforts. Additional information is needed to characterize the sentence contexts in which third-person subjects emerge and how subject diversity changes over time.

## A Descriptive Study of Subject Diversity in Simple Sentences

The second goal of this review article is to provide a detailed description of subject diversity in a sample of toddlers at risk for SLI. The primary objectives are to determine if subject diversity in third-person sentences emerges in a predictable developmental sequence and if toddlers at the greatest risk for persistent SLI show late emergence of third-person subjects. Subject diversity was explored in three simple sentence types.

The first and simplest sentence type is a state predicate with a subject and no lexical verb (cf. Rispoli, 2019). A state predicate ascribes some property to the sentence subject such as its location encoded with a prepositional phrase (PP; e.g., *The hat is on*) or an attribute encoded by an adjective phrase (AP; e.g., *The pizza is hot*), and in mainstream American English, a copula BE form is obligatory. Childlike versions of this sentence type emerge in Brown's (1973) Stage I without the copula BE, as word combinations such as *hat on* or *pizza hot,* more commonly referred to as semantic relations entity–location and entity–attribute, respectively. These childlike sentences have a single argument, the sentence subject, and take the form of S_NP_-(copula)-PP and S_NP_-(copula)-AP, respectively. Because the copula may or may not appear in these early sentences, they are characterized as having copula contexts. Rispoli (2019) demonstrated that argument diversity emerged in copula contexts prior to other simple sentence types and that simple state predicates are later incorporated into more complex SV and SVO sentences.

The second sentence type, SV sentences, is made up of a subject NP and an intransitive verb and takes the form S_NP_-VP. Because SV sentences have lexical verbs that are necessary for characterizing events, they are viewed as more complex than sentences with a copula BE context. Subject diversity in SV sentences may be greater than that in SVO sentences because the semantic roles of intransitive verbs are more diverse. Recall some intransitive verbs can have animate agents as subjects (e.g., *baby drinking*), whereas others have inanimate themes in subject position (e.g., *cake baking; pizza get hot; tower fall down*). Intransitive verbs that promote the themes to the subject position of SV sentences are known as unaccusatives (Reinhart, 2003). The third sentence type, SVO sentences, contains a subject NP, a transitive verb, and a direct object NP (e.g., *pig eating food; baby have hair*). SVO sentences are viewed as the most complex simple sentence type because transitive verbs require two obligatory arguments.

Following Rispoli (2019), we hypothesized that subject diversity in third-person sentences would be influenced by the complexity of the simple sentence type and appear in a developmental sequence: in one-argument copula contexts first, followed by one-argument SV sentences, and finally in two-argument SVO sentences. Based on the developmental patterns reported by Hadley et al. (2018) and McKenna and Hadley (2014), we also expected that toddlers with the greatest risk for persistent SLI, those with severe grammatical delays at 36 months, would have limited sentences with third-person subjects well beyond 30 months of age.

### The Archival Database: Participants and Language Samples

Archival longitudinal language samples from 22 participants obtained at 30, 33, and 36 months were used for this descriptive study. The language samples were originally gathered to study the emergence of tense/agreement morphemes in children at risk for SLI and those with low-average language development (Hadley & Holt, 2006; Hadley & Short, 2005). The Language Development Survey (Achenbach & Rescorla, 2000) was used to recruit toddlers to the study. Only children with expressive vocabulary abilities at or below the 40th percentile and whose families spoke only English in their homes were invited to participate. Families were primarily White, non-Hispanic with some college education or a 4-year degree. None of the children had a history of neurological, emotional, or behavioral impairments or of recurrent episodes of otitis media. All children had appropriate oral–motor abilities as demonstrated by a functional screening during the first visit (e.g., blow bubbles, suck water through straw, give kisses). All children also had normal hearing based on an evaluation by a clinically certified pediatric audiologist as part of the initial visit.

At study entry, 16 children were characterized as at risk for SLI (10 boys, six girls), and six had low-average language abilities (four boys, two girls) based on a comprehensive assessment. At-risk classifications were based on the presence of two or more risk factors (Hadley & Short, 2005): (a) a score below the 16th percentile on the receptive portion of the Test of Early Language Development–Third Edition (Hresko et al., 1999) or the Preschool Language Scale–Third Edition (Zimmerman et al., 1992); (b) a score below the 16th percentile on the CDI vocabulary checklist for age and sex; (c) a score below the 16th percentile for MLU (Miller & Chapman, 1981); (d) a family history of speech, language, or learning disabilities (Rice et al., 1998); and (e) a treatment history of language intervention prior to recruitment into this study. Children were identified as low average if they had CDI vocabulary scores below the 50th percentile for age and sex and no more than one risk factor.

At 36 months of age, average nonverbal cognitive abilities were determined using the Figure Ground and Form Completion subtests of the Leiter International Performance Scale–Revised (Roid & Miller, 1997). Expressive grammar abilities were also assessed with the IPSyn. IPSyn *z* scores were computed for 100 complete and intelligible utterances using the 36-month means and standard deviations from Scarborough's (1990) reference data. For details about the individual participants, measures, and reliability, refer to Hadley and Short (2005) and Hadley and Holt (2006).

For the current study, children were divided into three outcome groups based on the archival IPSyn *z* scores at 36 months of age. Children with low-average grammatical abilities (*n* = 8; six boys, two girls) had IPSyn *z* scores ranging from 0.01 to −0.99. This group included five of the six children initially identified with low-average language abilities and three toddlers originally classified as at risk whose language abilities had improved over time. Children with mild/moderate grammatical delays (*n* = 7; three boys, four girls) had IPSyn *z* scores ranging from −1.18 to −1.81. This group included one child initially identified with low-average language abilities and six at-risk toddlers. Finally, children with severe grammatical delays (*n* = 7; five boys, two girls) had IPSyn *z* scores ranging from −2.73 to −3.82 and had all been originally identified as at risk for SLI. IPSyn *z* scores falling below the 1st percentile placed these children at high risk for persistent SLI. It is also important to note that there was a substantial gap in IPSyn *z* scores between the minimum score of the mild/moderate delay group (−1.81) and the maximum score of the severe delay group (−2.73).

Means and standard deviations for traditional language sample measures at 36 months are presented in Table 1 for each outcome group. On average, children in all three groups produced more than 250 complete and intelligible utterances in 40 min of language sampling. Mean performance for the low average group was characterized by 90% intelligibility, an MLU above 3.00, and production of nearly 200 different words in spontaneous speech. Mean performance for the mild/moderate delay group was 88% intelligibility, an MLU above 2.50, and production of more than 150 different words in spontaneous speech. Finally, mean performance for the severe delay group was 77% intelligibility, an MLU above 2.00, and production of more than 125 different words in spontaneous speech. The group averages for MLU at 36 months place the groups in Brown's (1973) Stages IV, III, and II, respectively. These measures demonstrate that all children had the prerequisite abilities to produce one-argument sentences, namely, diverse expressive vocabularies and the ability to combine words regularly, by the end of the study.

**Table 1.** Traditional language sample measures by outcome groups at 36 months.

Language sample measures	*n*	Min	Max	*M*	*SD*
Low average	8				
C&I utterances		243.00	593.00	365.25	100.72
Intelligibility		0.82	0.97	0.91	0.05
NDW		157.00	238.00	197.88	26.21
MLUm		2.77	3.72	3.14	0.38
MLU *z* score		−0.56	0.81	−0.03	0.55
IPSyn *z* score		−0.99	0.01	−0.47	0.40
Mild/moderate delay	7				
C&I utterances		231.00	441.00	318.14	85.44
Intelligibility		0.78	0.93	0.88	0.06
NDW		121.00	204.00	171.00	32.09
MLUm		2.03	3.28	2.65	0.43
MLU *z* score		−1.63	0.17	−0.73	0.62
IPSyn *z* score		−1.81	−1.18	−1.57	0.23
Severe delay	7				
C&I utterances		138.00	390.00	272.57	82.52
Intelligibility		0.56	0.89	0.77	0.12
NDW		81.00	166.00	126.14	34.95
MLUm		1.53	2.42	2.02	0.30
MLU *z* score		−2.35	−1.07	−1.65	0.43
IPSyn *z* score		−3.82	−2.73	−3.21	0.36

*Note.* C&I utterances = number of complete and intelligible utterances, excluding abandoned, interrupted, nonverbal, imitative, and routine productions in a language sample; NDW = number of different words; MLUm = mean length of utterance in morphemes; IPSyn = Index of Productive Syntax.

Conversational language samples were gathered during parent–toddler free-play with a standard set of toys in a university play-room. Samples were obtained on two different days within a 2-week period. With two exceptions, two 20-min parent–child language samples were available for analysis for each child. The two samples were combined into a single transcript to compute the measures of interest. Participant 1226 had only one 20-min sample at 30 months, and none were available for participant 1109 at 33 months.

### Coding Scheme and Coding Procedures

Three types of simple sentences were of primary interest: state predicate (i.e., copula contexts), SV, and SVO sentences. These three sentence types reflect the presence or absence of a lexical verb and the number of arguments required by the lexical verb. Subjects and verbs were coded only in active declarative sentences. An active declarative sentence is a sentence with an explicit subject–predicate relation, in the active voice, with no structural movement of auxiliaries or *wh*-pronouns (Rispoli & Hadley, 2001; Rispoli et al., 2018). The Appendix includes further information on operational definitions, coding procedures, and examples of child utterances.

Subjects and verbs were coded only in spontaneous, complete, and intelligible utterances. Verbs were coded in all syntactic word combinations and sentence types (e.g., questions, imperatives, declaratives) and in both main and embedded clauses. Third-person subjects were coded only in active declarative sentences.

The coding conventions for third-person subjects were aligned with the second and third steps of the sentence-focused framework (Hadley, 2014). Because state predicates (e.g., *hat on, hands wet*) contain one of the fundamental building blocks of SV and SVO sentences, an overt subject, they were associated with the second developmental step. Third-person subjects appearing in S_NP_-(copula)-PP and S_NP_-(copula)-AP sentences were coded as [2:3N] for noun subjects and [2:3P] for pronoun subjects. Because subject–lexical verb combinations are associated with the third step of the sentence-focused framework, third-person subjects in SV and SVO sentences were coded as [3:3N] for noun subjects and [3:3P] for pronoun subjects.

Intransitive verbs were coded as [V:I] (e.g., *baby sleep*) and transitive verbs were coded as [V:T] (e.g., *I want that*) in all active declarative sentences, regardless of the person/number of the subject. This also allowed us to examine children's verb diversity in SV and SVO sentences with the earliest subject type, first-person singular *I* for comparison (Hadley et al., 2018).

Additional verb codes were used to exclude complex sentences from further analysis, including codes for infinitival complements, serial verb constructions, finite clause complements, nonfinite clause complements, and other complex sentence types. This decision to exclude complex sentences ensured that subject diversity was examined in comparable structures for children in all groups. The Appendix provides operational definitions and multiple examples of each code. Complex sentence types were made up primarily of *I*-subjects with the catenatives *gonna, wanna*, and *gotta* that take infinitival complements.

The number of different verbs in *I*-sentences (verb diversity) and the number of different subjects (subject diversity) in each third-person sentence type were computed automatically using Systematic Analysis of Language Transcripts software (Miller & Iglesias, 2017). Lexical variations of the same noun were counted as a single type (e.g., *dog, doggy =* 1 noun type, *dog*). Similarly, developmental errors on subject pronouns were not allowed to inflate the number of different subjects. That is, if a child produced both *he* and *him* as a subject pronoun, only *he* was counted. All forms of regular and irregular verb inflections were counted as a single type (e.g., *go, going, goes, went* = 1 verb type, *go*). A coding convention (e.g., doggy|dog, went|go, me|I, him|he) was used so that the subject and verb diversity measures could be generated automatically.

First, all sentences with verb codes associated with complex sentences were excluded from the analysis set. Then, we searched for specific SV combinations and saved the sets of sentences for each child to separate transcripts. For example, utterances containing both [3:3N] and [V:I] were saved as new transcripts with filenames for sentences with noun subjects and intransitive verbs were saved with the prefix NVI (noun verb intransitive), whereas utterances containing both [3:3N] and [V:T] were saved as new transcripts with the prefix NVT (noun verb transitive). Subject and verb diversity measures were then computed by exploring transcripts for the subject and verb codes and outputting the number of different words associated with these codes to a reference database.

All transcripts were coded by a member of the research team, after completing a training program in linguistic coding, computerized quizzes, and multiple practice samples. Initial coding was completed by one coder and checked by a second coder for every transcript (100%). As part of the coding process, coders used a series of computerized searches to check all codes for errors of commission as well as to check all utterances without subject and verb codes for errors of omission. Coders also flagged difficult utterances for discussion during lab meetings. All coding questions were resolved, and errors were corrected prior to conducting the final analyses.

### Descriptive Results

**Figure 2.**
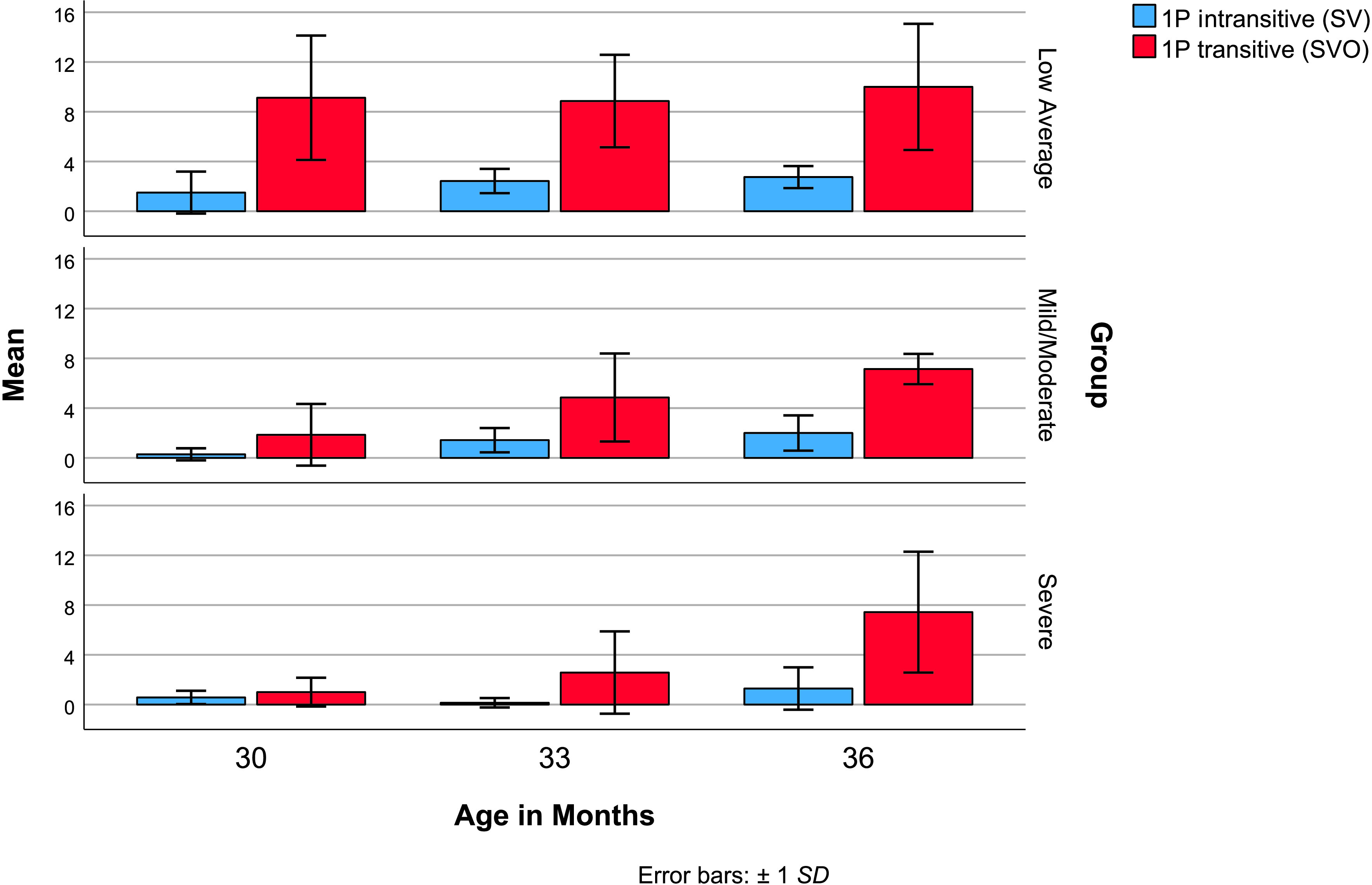
Verb diversity with first-person singular *I*-subjects. SV = subject–verb sentence; SVO = subject–verb–object sentence.

To demonstrate that all children were capable of producing SV combinations by 36 months, verb diversity in sentences with *I*-subjects was explored first. Table 2 reports descriptive statistics for verb diversity in SV and SVO *I*-sentences. The average child in the low average group produced *I*-subjects with more than 10 different verbs from 30 months on (see Table 2). All children in the low average group produced *I*-subjects with two or more different verbs by 30 months, all children in the mild/moderate delay group met this criterion by 33 months, and all children in the severe delay group met this criterion by 36 months. For all groups at all ages, the majority of *I*-subjects occurred with transitive verbs in SVO sentences. The most common transitive verbs were internal states *want* and *need*; the possessive state *got* (meaning *have*); and general all-purpose (GAP) actions *get* (meaning obtain), *put,* and *do* (cf. Rice & Bode, 1993). These verbs were used by 10 or more children in SVO sentences with *I*-subjects at 36 months (see Supplemental Material S1 for verbs by subject type). Figure 2 illustrates developmental change in verb diversity for *I-*sentences with intransitive and transitive verbs.

**Table 2.** Verb diversity with *I*-subjects by outcome group and measurement point.

Measures		30 months				33 months				36 months			
		Min	Max	*M*	*SD*	Min	Max	*M*	*SD*	Min	Max	*M*	*SD*
Low average	*n* = 8^a^												
Intransitive (SV)		0	4	1.50	1.69	1	4	2.43	0.98	1	4	2.75	1.50
Transitive (SVO)		2	15	9.13	5.00	5	13	8.86	3.72	5	20	10.00	9.13
Total		2	15	10.63	5.71	7	17	11.29	4.27	8	23	12.75	5.09
Mild/moderate delay	*n* = 7												
Intransitive (SV)		0	1	0.29	0.49	0	3	1.43	0.98	1	5	2.00	0.29
Transitive (SVO)		0	7	1.86	2.48	1	10	4.86	3.53	6	9	7.14	1.86
Total		0	7	2.14	2.54	2	11	6.29	3.55	7	13	9.14	2.04
Severe delay	*n* = 7												
Intransitive (SV)		0	1	0.57	0.53	0	1	0.14	0.38	0	5	1.29	1.70
Transitive (SVO)		0	3	1.00	1.15	0	9	2.57	3.31	2	15	7.43	4.86
Total		0	4	1.57	1.62	0	10	2.71	3.64	2	17	8.71	5.99

*Note.* SV = subject–verb sentence; SVO = subject–verb–object sentence.

a At 30 months, one participant had only 20 min of data; at 33 months (*n* = 7) because one participant had missing data.

**Figure 3.**
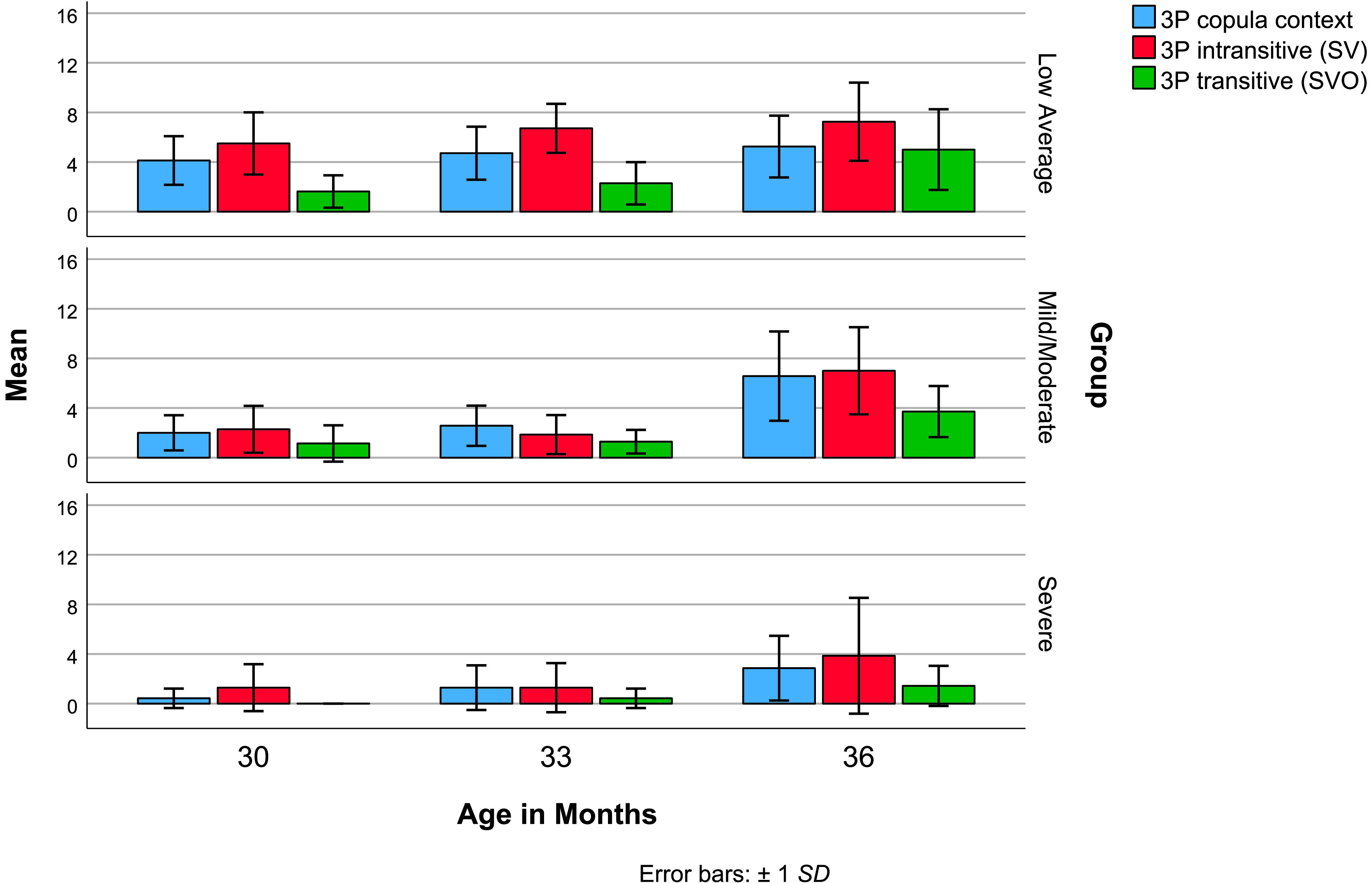
Third-person subject diversity by sentence type. SV = subject–verb sentence; SVO = subject–verb–object sentence.

Given the limited number of sentences with third-person subjects, noun and pronoun subjects were analyzed together. Table 3 reports descriptive statistics for subject diversity in copula contexts (i.e., entity–location, entity–attribute), in SV sentences with intransitive verbs, and in SVO sentences with transitive verbs. For the average child, third-person subjects were most diverse with intransitive verbs and copula contexts and least diverse with transitive verbs at all measurement points (see Figure 3). The average child in the low average group produced five or more different third-person subjects from 30 months on (see Table 3). At 30 months, all eight children in the low average group produced sentences with at least two different third-person subjects in one-argument copula contexts and SV sentences. In contrast, only 29% and 14% of children in mild/moderate and severe delay groups, respectively, had reached this same milestone. By 36 months, 88% and 86% of the children in the low average and mild/moderate delay groups produced two different third-person subjects across all three simple sentence types. However, only 29% of the children in the severe delay group met this criterion. In fact, third-person subjects were completely absent for four children in the severe delay group at 30 months of age, for one child at 33 months, and for two children at 36 months.

**Table 3.** Third-person subject diversity with verb types by outcome group and measurement point.

Measures		30 months				33 months				36 months			
		Min	Max	*M*	*SD*	Min	Max	*M*	*SD*	Min	Max	*M*	*SD*
Low average	*n* = 8^a^												
Copula context		2	7	4.13	1.96	2	7	4.71	2.14	2	9	5.25	2.49
Intransitive (SV)		3	11	5.50	2.51	3	9	6.71	1.98	3	10	7.25	3.15
Transitive (SVO)		0	4	1.63	1.30	0	5	2.29	1.70	1	10	5.00	3.25
Total		7	18	11.25	4.13	10	20	13.71	3.86	8	27	17.50	7.91
Mild/moderate delay	*n* = 7												
Copula context		0	4	2.00	1.41	1	5	2.57	1.62	3	14	6.57	3.60
Intransitive (SV)		1	5	2.29	1.89	0	4	1.86	1.57	1	10	7.00	3.51
Transitive (SVO)		0	3	1.14	1.46	0	3	1.29	0.95	2	8	3.71	2.06
Total		1	12	5.43	4.16	1	10	5.71	2.93	11	32	17.29	6.97
Severe delay	*n* = 7												
Copula context		0	2	0.43	0.79	0	5	1.29	1.80	0	6	2.86	2.61
Intransitive (SV)		0	4	1.29	1.89	0	5	1.29	1.98	0	13	3.86	4.67
Transitive (SVO)		0	0	0.00	0.00	0	2	0.43	0.79	0	4	1.43	1.62
Total		0	6	1.71	2.63	0	9	3.00	3.51	0	18	8.14	6.87

*Note.* SV = subject–verb sentence; SVO = subject–verb–object sentence.

a At 30 months, one participant had only 20 min of data available, and at 33 months (*n* = 7), one participant had missing data.

At 36 months, the most common third-person subjects were pronouns *it* and *he*. These pronouns were used by 10 or more children across all three sentence types. Only three noun subjects were produced by five or more children: *boy, baby,* and *car*. The distribution of noun subjects in copula contexts was also noteworthy. Children produced more different inanimate noun subjects than animate noun subjects in copula contexts (i.e., 27 vs. 7; see Supplemental Material S2 for third-person subjects by sentence type). The most common intransitive verbs appearing with third-person subjects were the GAP verbs *go, come,* and *get* (meaning *become*) and the more lexically specific verb *fall*. These intransitive verbs were also used by 10 or more children in SV sentences across the combination of noun and third-person pronoun subjects (see Supplemental Material S1 for verbs by subject type).

### Discussion

This study provided new evidence about expectations for sentence diversity. By 30–32 months, most typically developing children produce diverse sentences, including both SV and SVO sentence types and both first- and third-person subject types (Hadley et al., 2018; Klee & Gavin, 2010). The descriptive analyses from this study revealed that different subjects and verbs are found in early SVO and SV sentences. For all groups, *I*-subjects were primarily found in early SVO sentences, whereas third-person subjects appeared more often in early SV sentences. By studying children developing language at very slow rates, these new patterns in sentence development were made obvious. Interestingly, Figure 1 also displays this same pattern. For this child at 24 months, first-person *I*-subjects appeared exclusively with transitive verbs, and third-person subjects appeared exclusively with intransitive verbs. An important clinical implication follows. First, we should not assume that children who produce diverse SVO sentences with *I*-subjects also produce diverse SV sentences. Second, to increase third-person subject diversity, we may also need to build up the intransitive verb lexicon.

Returning to the primary objectives of the pilot study, subject diversity was indeed influenced by the number of arguments in simple sentences. As third-person subjects emerged, they were more diverse in one-argument copula contexts and with intransitive verbs than with two-argument transitive verbs, providing partial support for our hypothesis. Average third-person subject diversity with intransitive verbs exceeded that of transitive verbs for all groups at all measurement points. We also hypothesized that third-person subjects would be more diverse in copula contexts compared to intransitives when they emerged; however, an early advantage for copula contexts was not apparent. Our prediction was based on Rispoli's (2019) study of children with low-average language abilities. Rispoli observed greater subject diversity in copula contexts at 24 months when his participants had a mean MLU of 1.86. Three months later, when the participants' mean MLU had increased to 2.00, subject diversity was greatest with intransitive verbs. It is likely that the low average group, with a mean MLU of 2.31 at the initial 30-month measurement point, was already beyond this shift, but the two delay groups were not. The most likely explanation for the different pattern of findings relates to differences in the predicate complexity of the SV sentences. SV sentences in Rispoli (2019) were required to have result phrases (e.g., *tower fall down*, *it get out*). In the current study, a greater variety of intransitive sentences were allowed, including those without result phrases (e.g., *he hide, it fall*) as well as activity predicates (e.g., *baby sleeping*).

Finally, we predicted that toddlers with severe grammatical delays would have limited third-person subject diversity at 30 months and beyond. Indeed, third-person subjects appeared latest for this group, and third-person subjects were absent for two of seven children in the severe delay group at 36 months. It is important to reiterate that these children were producing diverse sentences with the subject *I.* They also used third-person pronouns such as *it* and *this* as direct object NPs. Nevertheless, third-person subject NPs were absent. To illustrate this phenomenon, the simple sentences of participant 1122 are presented below. Despite community-based early intervention from 17 to 36 months of age, this child's IPSyn score fell −3.18 *SD* below the mean for his age. Information obtained from parent report and language sampling at 36 months revealed several indicators of readiness for third-person sentences. His parents reported he used 632 total words on the CDI, including all 103 verbs. In his language sample, he combined words regularly obtaining an MLU of 1.92, used 106 different words including 18 different verbs, and produced 10 different SV combinations with transitive verbs. Yet, this child did not produce any sentences with third-person subjects in 299 complete and intelligible utterances.

1. I want mail.me want *NP please.
2. we want (this) this.
3. me need this.me need mail.me need Name.
4. me like that snake.
5. me see it.
6. me eat it.
7. me do carwash.
8. me wash *NP *P carwash.
9. I put *NP right here.
10. me go up there.

Taken together, the findings from these descriptive analyses have implications for monitoring risk for SLI with young children. We propose that the absence of third-person subjects at 30 months should raise concern and warrant monitoring of subject diversity from 30 to 36 months (see Hadley, 2014; McKenna & Hadley, 2014), just as the absence of verbs at 24 months raises clinical concern and warrants monitoring of subsequent verb growth from 24 to 30 months (Hadley et al., 2016; Olswang et al., 1998). Moreover, because later emergence and low diversity of third-person subjects were associated with severe delays on the IPSyn at 36 months, the absence of third-person subjects at 36 months may elevate a child's risk for persistent SLI.

## General Conclusions, Limitations, and Future Directions

The capacity to combine many different subjects and verbs flexibly is what enables young children to express complex ideas precisely, moving beyond general messages about their wants, needs, and desires to comments and descriptions about interesting events. The program of research reviewed in this review article demonstrates that measures of sentence diversity hold promise for characterizing differences in this capacity. The measures can be used to characterize differences between children and developmental changes over time. These measures are not intended to replace comprehensive measures of grammar. Rather, the intent is to identify treatment targets for intervention that have the potential to stimulate broader aspects of grammatical development.

In light of the limited subject diversity observed in the descriptive study for toddlers with severe grammatical delays, we propose that subject diversity should be prioritized as a treatment target in early language interventions and evaluated in future clinical research. From our perspective, the number of different words a child produces in subject position is an indicator of the strength of the subject constituent in the child's grammar (cf. Hadley, Rispoli, Holt, Papastratakos, et al., 2017; Rispoli et al., 2018). Opportunities to comprehend and produce sentences about third-person subjects are crucial for strengthening the representation of subject in the child's grammar because only these sentences can be filled with an infinite number of different nouns and variety of pronouns (e.g., *it, that, those, he, she, they*). Sentences about the speaker or listener are limited to the subject pronouns *I* and *you;* therefore, these sentence types may be less useful for strengthening the child's representation of subject. We suspect the development of a strong representation of sentence “subject” may be a watershed event for English-speaking toddlers. In typical development, the first tense/agreement morphemes appear in sentences with third-person singular subjects (i.e., copula *is,* third-person singular –*s*; Hadley et al., 2014; Rispoli, Hadley, & Holt, 2009; Rispoli et al., 2012). More adultlike forms of negation and questions also depend upon the presence of sentence subjects (Brown, 1973; Miller, 1981). These more advanced structures emerge rapidly between 2 and 3 years of age. Given the essential relationship of subjects to these structures, it is not surprising that the children with limited subject diversity in this study had severe grammatical delays on the IPSyn at age 3 years.

Some readers may have reservations about measures of emergence and diversity obtained from language samples. When a grammatical structure is absent or has limited diversity, is this a true indicator of the child's language abilities or were there insufficient opportunities to produce the structure? To address this concern, we include a number of different objects in our toy sets and collect language samples with examiners who intentionally shift the conversation toward these objects to create opportunities for diverse sentences (cf. Hadley et al., 2014; Oetting & Hadley, 2017). We also believe the developmental trends for the sentence diversity measures across different longitudinal study cohorts (Hadley, Rispoli, Holt, Papastratakos, et al., 2017; Rispoli, 2018) and for different ability levels in Hadley et al. (2018) and in the current study reduce this concern. That is, the consistent evidence of developmental trends increases the likelihood that limited sentence diversity does indeed reflect less advanced sentence development.

A major limitation of the studies reviewed is the demographic homogeneity of the participants. All studies focused exclusively on monolingual, English-speaking families. Participants have been drawn primarily from White, non-Hispanic, college-educated families. Thus, the developmental expectations for sentence diversity provided here should be applied cautiously to children from culturally, linguistically, and economically diverse backgrounds. At the same time, the sentence is the most fundamental unit of syntax, so efforts to assess and strengthen the representation of basic sentence structure in the underlying grammar are relevant to child speakers of all dialects of English. The principles of the sentence-focused framework could also be adapted for other languages, and future research could explore whether the relatively simple measure of subject diversity identifies similar patterns of subject expansion in other languages (i.e., first-person singular, third-person singular, plural).

The findings of this pilot study have also led to a new appreciation for simple state predicates as targets of intervention, specifically the semantic relations entity–location and entity–attribute. Table 4 presents the interface between the syntactic structures explored in this study and semantic meaning based on Ramchand's (2008) theory of event structure. Clinicians could target noun subjects in entity–location and entity–attribute relations once children have acquired a core vocabulary of nouns and a set of early developing prepositions and adjectives. Note that these entities can be either animate (e.g., *cow in*) or inanimate (e.g., *hat on*). In fact, it may be helpful to emphasize inanimate entities to contrast with the animate agent and experiencer roles associated with early *I*-sentences (e.g., *I do it*, *I want that*). As children acquire intransitive verbs that encode simple change of motion or state, these verbs could be added into the simple state predicates. The addition of these verbs will transform a semantic relation into an SV sentence. Recall that the most common intransitive verbs used by the children in this study were *go, come, fall,* and *get*. *Go, come,* and *fall* specify the directional path along which the entity moves to a new location (e.g., *cow go in; hat come off*). *Get* specifies the change from one state to another (e.g., clean ➔ dirty; *hands got dirty*). The general meanings of GAP verbs allow them to combine readily with a wide variety of noun subjects. Work is currently underway to evaluate the efficacy of this proposed target sequence.

**Table 4.** Hierarchy of simple sentence complexity.

Sentence structure	Event structure	Childlike examples within adult sentences
Copula context S_NP_-(cop)-PP S_NP_-(cop)-AP	State predicate Entity–location Entity–attribute	*The* ***cow** is **in**. The **hat** is **off**. The **tower** is **down**.* *My **hands** are **clean**. The **pizza** is **hot**.*
SV sentence S_NP_-VP-PP S_NP_-VP-AP S_NP_-VP	Dynamic event Entity-GO-location Entity-BECOME-state Entity-activity	*The **cow go**es **in**. The **hat*** ***came*** ***off**. The **tower fell down**.* *My **hands** are **get**ting **clean**. The **pizza** is **get**ting **hot**.* *The **baby** is **sleep**ing. **He***'*s **play**ing.*

*Note.* S_NP_ = subject noun phrase; PP = prepositional phrase; AP = adjective phrase; SV = subject–verb.

Future investigations should also examine diversity in the semantic roles associated with subject position in SV and SVO sentences. Nearly 50 years ago, Bowerman (1973) described increasing diversity in the semantic roles of subject position between Brown's (1973) early (MLU < 1.50) and late (MLU 1.50–2.00) Stage I. In early Stage I, she characterized the grammatical function of subject as strongly associated with the semantic function “agent” referring to animate beings and vehicles capable of initiating actions. Only in late Stage I did “nonagentive” or inanimate subjects emerge, along with verbs associated with inanimate, nonagentive subjects. These nonagentive subjects are associated with unaccusative intransitive verbs (e.g., *break, fall, open, roll, spill, stop*) that promote the affected object to the subject NP position (Reinhart, 2003), and children with SLI have been shown to have difficulty producing subjects with unaccusative intransitives (Fletcher et al., 2012; Grela & Leonard, 1997). Children with SLI may need more time and/or more support to dissociate the grammatical function subject from the more prototypical semantic roles of agent and experiencer.

In closing, the emergence of diverse, simple sentences follows a similar developmental pattern for toddlers with low-average language abilities and toddlers at risk for SLI. Although all toddlers with low-average language abilities produced multiple sentences with third-person subjects by 30 months of age, third-person subjects were absent or limited for most children with severe grammatical delays at 36 months. To promote linguistic flexibility and support subsequent grammatical development, strategies to strengthen the representation of the sentence subject in children's developing grammars are recommended.

## Supplementary Material

10.1044/2020_JSLHR-20-00031SMS1 Supplemental Material S1 Verbs by subject type.

10.1044/2020_JSLHR-20-00031SMS2 Supplemental Material S2 Third-person subjects by sentence type.

10.1044/2020_JSLHR-20-00031SMS3 Supplemental Material S3 Presentation video from the Research Symposium at the 2019 Annual Convention of the American Speech-Language-Hearing Association held in Orlando, FL.

## Footnote

1 Reference to *I*-subjects and *I*-sentences includes all child first-person singular subjects, regardless of whether it was produced correctly or developmental errors were observed (e.g., *me/I; my/I; Child's Name/I*).

## Appendix

### Coding Scheme for Assessing Third Person Subject Diversity in Simple Sentences

**Table:**

Only spontaneous, complete, and intelligible utterances are coded for third-person subjects and lexical verbs. **Lexical verbs** are coded in all syntactic combinations and sentence types (e.g., questions, imperatives, declaratives) and in both main and embedded clauses. **Third-person subjects** are coded only in active declarative sentences. Active declarative sentences have an explicit subject–predicate relation, in the active voice, with no syntactic movement of auxiliaries or *wh*-pronouns.		
**Verb Type**	**3rd-Person Pronoun Subject**	**Noun Subject**
**Copula Context** refers to a state predicate ascribing a location or attribute to the sentence subject. These predicates have the form: S-(copula)-PP or S-(copula)-AP. The copula is not required; it will often be omitted. S-(copula)-NP are excluded (e.g., it's/that's a NP, or NP is mine/yours).	The subject code [2:3P] is used with third-person pronoun subjects with a prepositional phrase or an adjective phrase. this[2:3P] on. it[2:3P] off. it[2:3P] dirty. that[2:3P]/'s yellow.	The subject code [2:3N] is used with noun subjects with a prepositional phrase or an adjective phrase. eye[2:3N] off. teeth[2:3N] out. horse[2:3N] too big. Danny[2:3N]/'s clean.
	The subject code [3:3P] is used when a third-person pronoun subject is combined with a verb phrase.	The subject code [3:3N] is used when a noun subject is combined with a verb phrase.
**Intransitive [V:I]** verbs are the foundation of SV sentences. They do not require/allow a direct object noun phrase.	this[3:3P] go[V:I] home. it[3:3P] come[V:I] off. it[3:3P] don't fit[V:I].	the pig[3:3N] go[V:I]/ing in. a ball[3:3N] come[V:I]/ing out. tower[3:3N] fall[V:I] down.
**Transitive [V:T]** verbs are the foundation of SVO sentences. They require a direct object noun phrase.	she[3:3P] drink[V:T]/ing juice. he[3:3P] feed[V:T]/ing pig/s. she[3:3P] like[V:T] him. him|he[3:3P] need[V:T] eyes.	the girl[3:3N] do[V:T]/ing it. Elmo[3:3N] touch[V:T] it. farmer[3:3N] want[V:T] a pumpkin. baby[3:3N] have[V:T] hair.

**Table:**

All lexical verbs receive a verb code. However, sentences with two or more verbs are excluded from the analyses of sentence diversity in simple sentences. The following main verb codes are used to exclude these sentences. Because third-person subjects are rare with these verb types, examples with first-person subjects are included. Noun subjects in embedded clauses are coded, but these subjects are excluded from the analyses of subject diversity in simple sentences.		
**Verb Type**	**1st- and 3rd-Person Pronoun Subjects**	**Noun Subjects**
**Infinitival Verb Complement [V:toV]** is coded when the verb takes an infinitival form of a verb *(to [verb])* as its complement. This includes catenatives wanna, gonna, hafta, gotta, as well as try/need/like to V.	I wanna[V:toV] turn[V:T] this off. I need[V:toV] to get[V:T] the hotdog (uh) out. I want[V:toV] *to try[V:toV] *to do[V:T] this. he[3:3P] has[V:toV] to stop[V:I]. he[3:3P]/'s gonna[V:toV] comb[V:T] my hair. he[3:3P] *is try[V:toV]/ing *to see[V:T] me. her|she[3:3P] gotta[V:toV] do[V:T] this.	daddy[3:3N]/'s go[V:toV]/*ing *to fall[V:I] down. now the baby[3:3N] has[V:toV] to lay[V:I] down. Santa[3:3N] have[V:toV] *to bring[V:T] me some barbie/s. this block[3:3N] *is gonna[V:toV] fall[V:I]. the bubble[3:3N]/*s have[V:toV] *to go[V:I] in *the slide. the caboose[3:3N] has[V:toV] to be at this end.
**Serial Verb and Aspectual Verbs [V:V]** Serial verb combinations begin with directional verbs *go* and *come*. Aspectual verbs such as *start, stop, keep* have gerund complements V–*ing*. Aspectual verbs refer to the initiation, continuance, or termination of an event (e.g., *start running, stop running, keep running*).	I go[V:V] get[V:T] the ball. I/'ll go[V:V] call[V:I]. (it cames) it[3:3P] keep[V:V]/3s come[V:I]/ing out.	
**Nonfinite Clausal Complement [V:NC]** This verb code is used when the complement of the verb should be a dependent clause with a new subject and nonfinite verb form.	that[3:3P] make[V:NC]/*3s the baby cry[V:I]. he[3:3P] want[V:NC]/*3s the car to go[V:I] in there.	
**Finite Clausal Complement [V:FC]** This verb code is used when the complement of the verb is a dependent clause with a new subject and finite verb form.	I thought[V:FC] she want/ed[V:NC] (those i* f*) it full. her|she[3:3P] can go[V:FC] anywhere her|she want/3s.	(um) a people[3:3N] says|say[V:FC] (s* s*) they *do not need[V:TS] this.
**Relative or Adjunct Clause [V:CX]** This verb code is used with complex sentences to indicate that the dependent clause is NOT a complement of the main verb. Code the main verb as V:CX when the dependent clause is a **relative clause** (a clause that starts with who, that, which, whose, where, when and modifies the preceding noun) OR an **adjunct clause** (a clause that adds extra information but is not necessary to the overall structure of the sentence).	(that on on) I can get[V:CX] (s* es*) the other stuff (on) on the train, so the train[E:3N] (can) can move[V:I].	
**Embedded 3rd-Person Noun Subject [E:3N]** This subject code will only be used when the noun subject appears in an embedded finite clause of a complex sentence.		that/'s where *the umbrella[3:3N] go[V:I]/3s. (that on on) I can get[V:CX] (s* es*) the other stuff (on) on the train, so the train[E:3N] (can) can move[V:I]. help[V:T] me because mrpotato[E:3N] *is in there.
